# Poisson‐event‐based analysis of cell proliferation

**DOI:** 10.1002/cyto.a.22620

**Published:** 2015-01-08

**Authors:** Huw D. Summers, John W. Wills, M. Rowan Brown, Paul Rees

**Affiliations:** ^1^ Systems and Process Engineering Centre, College of EngineeringSwansea UniversitySingleton ParkSwanseaSA2 8PPUnited Kingdom

**Keywords:** Key terms: cell proliferation, mitosis, poisson distribution, computer‐assisted image analysis, microscopy, correlation studies

## Abstract

A protocol for the assessment of cell proliferation dynamics is presented. This is based on the measurement of cell division events and their subsequent analysis using Poisson probability statistics. Detailed analysis of proliferation dynamics in heterogeneous populations requires single cell resolution within a time series analysis and so is technically demanding to implement. Here, we show that by focusing on the events during which cells undergo division rather than directly on the cells themselves a simplified image acquisition and analysis protocol can be followed, which maintains single cell resolution and reports on the key metrics of cell proliferation. The technique is demonstrated using a microscope with 1.3 μm spatial resolution to track mitotic events within A549 and BEAS‐2B cell lines, over a period of up to 48 h. Automated image processing of the bright field images using standard algorithms within the ImageJ software toolkit yielded 87% accurate recording of the manually identified, temporal, and spatial positions of the mitotic event series. Analysis of the statistics of the interevent times (i.e., times between observed mitoses in a field of view) showed that cell division conformed to a nonhomogeneous Poisson process in which the rate of occurrence of mitotic events, *λ* exponentially increased over time and provided values of the mean inter mitotic time of 21.1 ± 1.2 hours for the A549 cells and 25.0 ± 1.1 h for the BEAS‐2B cells. Comparison of the mitotic event series for the BEAS‐2B cell line to that predicted by random Poisson statistics indicated that temporal synchronisation of the cell division process was occurring within 70% of the population and that this could be increased to 85% through serum starvation of the cell culture. © 2015 The Authors. Published by Wiley Periodicals, Inc.

The study of cell proliferation is a staple cytometry technique which reports on the fundamental biology of the cell cycle and is a key indicator in pharmacological and toxicological assays 1. The processes of cell growth and division within heterogeneous populations can produce complex population dynamics 2, 3, 4. Thus, accurate, quantitative analyses of cell proliferation, which take full account of spatial and temporal variation of distinct population subgroups, are challenging. Methods for assessing cell proliferation can be organized according to their analytical complexity with the simplest approach being counts of total cell number and the most sophisticated providing complete tracking of cellular state and position through time‐lapse imaging.

Population level measures such as total cell mass or cell count provide relatively straightforward analyses of proliferation but are ultimately limited as they report on the outcome of biological processes (i.e., population size) rather than the processes themselves 5. They are also predominantly implemented as an end point assay and are thus limited to a single time point. At the other end of the spectrum, time lapse microscopy, and computational image processing provides full quantification of individual cells in space and time; however it is expensive to implement and requires a high level of experience and expertise 6. Between these extremes lies a collection of flow cytometry techniques which provide cell cycle or generational information through measurement of fluorescent molecular reporters 7, 8, 9, 10. These are implemented at the single cell level and so capture population heterogeneity, but they cannot capture the inter‐relations of cells in space and time as they are implemented as end point assays of randomized cell suspensions.

In this article, we present a microscopy technique in which time lapse imaging is used to identify division events as opposed to directly tracking the cells. This provides a measure of cell proliferation based on recognition of the binary division process and so economises the measurement task by only collecting information at those critical time points within the cell life cycle at which mitosis drives population growth. The technique therefore provides single cell resolution and mapping of spatiotemporal events but in a simplified implementation where image processing is made relatively straightforward by the single requirement to recognize mitotic cells; that is, cells only need to be identified when in a high image‐contrast mode as they lift off the growth surface and become spherical, prior to a division event. It is the measurement of an event sequence that is of primary importance here, as this provides data on the occurrence rate, *λ* of events and the intervals, Δ*t*, Δ*x*, and Δ*y* between them. Comparison can then be made to the expected values, as described by Poisson statistics 11. Agreement of the data with a predicted Poisson event series indicates that the underlying principles of this statistical process, namely random occurrence of events with no correlation to previous instances, are consistent with the observed behavior of the biological system. The technique therefore indicates whether there is spatial or temporal synchronicity within the cell population 12. Fitting of the intermitotic times according to Poisson statistics also provides a measure of the mean rate of event occurrence and the associated mean rate of cell growth, *γ*. Thus, the inter‐mitotic time can be quantified without the need to track individual cells from the point of their birth through to the creation of daughter cells.

We implement the technique using a JuLI™ microscope (NanoEn Tek, Seoul, Korea). This machine is designed to sit within a standard cell culture incubator and provides automated image acquisition over an extended period at user defined times. Its performance is of relatively low specification, with an optical resolution of just 1.3 μm. However, this is more than sufficient to detect mitotic cells and our results demonstrate how the low‐level demands of the Poisson analysis on the optics of the instrument allow a robust and detailed assessment of cell population proliferation dynamics.

## Materials and Methods

### In‐Vitro Cell Culture

Normal lung (BEAS‐2B) and lung carcinoma (A549) cell lines were purchased from ATCC® (product numbers CRL‐9609 and CLL‐185, respectively). Cultures were established under standard conditions (37 °C, 5% CO_2_, 95% humidity) in a growth medium consisting of glutamine containing Dulbecco's Modified Eagle's Medium (DMEM; D5796, Sigma‐Aldrich, UK), supplemented with 10% foetal bovine serum (FBS; 10270–106, Life Technologies, UK), 1% penicillin/streptomycin (15240–062, Life Technologies, UK). Prior to the initiation of experiments cultures were maintained for at least 72 h at ≤75% confluency by routine subculturing involving trypsinization (25300–054, Life Technologies, UK), centrifugation (185*g*, 5 min) and re‐seeding in T75 culture flasks (658175, Greiner Bio‐One, UK). For time‐lapse microscopy experiments, cell densities were determined using a Z1 series Coulter Counter and seeded into T25 flasks (690175, Greiner Bio‐One, UK). Cultures were then returned to the incubator for 12 h to facilitate attachment prior to initiation of imaging. Cell synchronization was achieved by serum starvation; initiated through replacement of the FBS containing medium with 99% DMEM/1% antibiotics.

### Time‐Lapse Microscopy and Image Acquisition

Time‐lapse microscopy was carried out using a JuLi™ Smart Fluorescent Cell Analyser automated microscope (NanoEn Tek, Seoul, Korea) equipped with a 4X objective and operating under the 20× digital zoom setting. Images were captured in bright‐field mode at 15 min intervals at 640 × 540 pixel resolution, for up to 48 h. During imaging, the portable microscope was situated inside the incubator to ensure optimal conditions for cell growth.

### Analysis Using Poisson Statistics

The analysis of cell proliferation is based on the measurement of a series of mitotic events. Consider the average rate at which mitoses occur, at a point in time that is captured by a single image frame. This is designated by the symbol, *λ* and is related to the number of cells present, *N*_cell_ (which is in turn related to the image field size) and the cell population growth rate, *γ* :

(1) $$\lambda =\gamma N_{cell}$$

(2) $$N_{cell}=N_{0}e^{\gamma t}$$

Where *N*_0_ is the number of cells at *t* = 0 and the rate of cell death is assumed to be negligible in comparison to the growth rate. Substituting for *N*_cell_ gives:

(3) $$\lambda ={\gamma N_{0}e}^{\gamma t}$$

The series of mitoses occurring over a given time period can be viewed as a specific example of a general class known as Poisson processes. These are characterised by a series of mitotic events, the occurrence of which is random from event to event but can be characterised by a constant, average rate of occurrence, *λ* over longer time scales. For such a Poisson event series the probability that a given interevent duration will be greater than the time variable, *τ* is described by:

(4) $$P\left(\Delta t>\tau \right)=e^{-\tau }$$

However, as shown by Eq. (3), in the case of cell proliferation *λ* is time dependent—as the cell population increases so will the average number of mitoses per unit time. In cases where *λ* is time‐dependent the event sequence forms a nonhomogeneous Poisson series 13. In this case Eq. (4) cannot be directly applied. However, in general, *λ* (*t*) will be a slowly varying function over the time scale of *τ* and so Eq. (4) will hold over limited time intervals. For the cell mitosis example we consider, *λ* (*t*) varies over 24 h timescales whilst the time interval between observed mitoses, *τ* is typically a few hours. We can therefore obtain the probability of finding an interevent spacing, *τ*, anywhere within a measurement period, *t*′ by using the average value of *λ* (*t*) between *t = 0* and *t = t*′, in Eq. (4).

From Eq. (3) the average rate, *Λ,* determined over a measurement period, *t'* is:

(5) $$\Lambda =\frac{1}{t^{'}}\int_{0}^{t^{}}\lambda \left(t\right)dt=\frac{N_{0}}{t'}[e^{\gamma t}‐1]$$

The probability distribution of the interevent duration, Δ*t*, over a series of duration *t*′, is thus:

(6) $$P\left(\Delta t>\tau \right)=e^{-\Lambda \tau }$$

Thus *Λ* can be determined from the measured mitotic event series using Eqs. (5) and (6) and used, together with a count of the cell number in the initial time frame (*N*_0_), to determine the cell growth rate, *γ*. Finally, the intermitotic time, *t*_IMT_ can be calculated using the relation, *t*_IMT_ *=* ln(2 *)/γ*.

## Results

### Time‐Series Analysis

**Figure 1:**
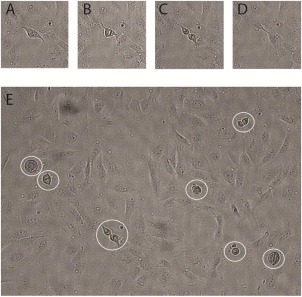
**A**–**D**: Representative time series, of reduced image field size, showing a single cell division event imaged at 15 min intervals; (**E**) full image field with mitotic cells highlighted with overlaid circles. All images are of BEAS‐2B cells seeded at a density of 2 × 10^5^ cells ml^−1^.

**Figure 2:**
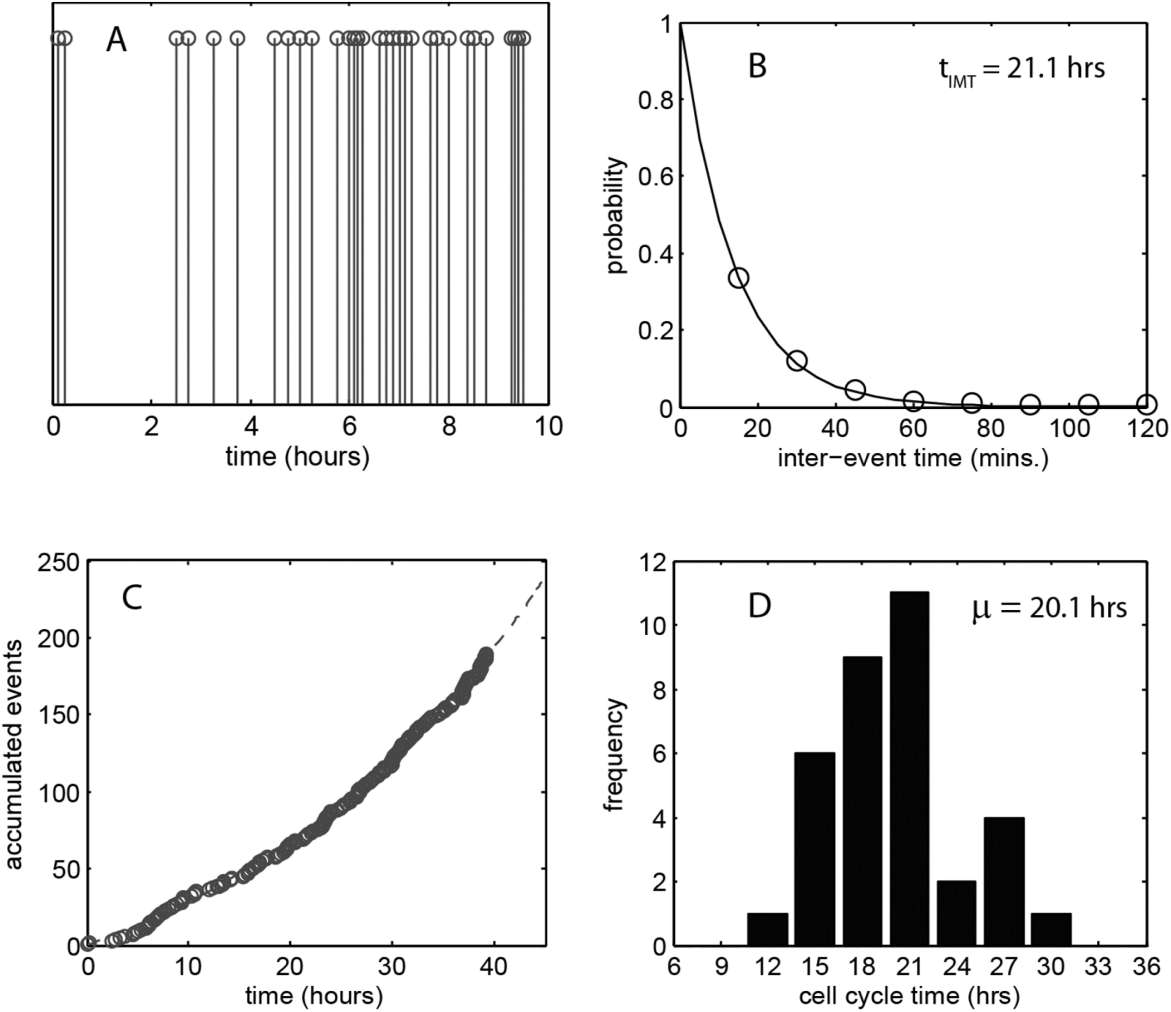
**A**: Time series of division events for an A549 cell culture, seeded at a density of 1.5 × 10^5^ cells ml^−1^. An expanded view of the first 10 h of a 40 h time‐lapse experiment is shown to ensure clear display of events. **B**: Probability distribution of the interevent time for all events within the 40 h period (circles – measurement data, solid line – statistical fit). The *t*_IMT_ value indicated is obtained by a best fit to the data assuming a nonhomogeneous Poisson process. **C**: Accumulated count of cell division events (circles) with a model prediction (dashed line) based on exponential population growth. **D**: Histogram of measured cell cycle time obtained from manual, frame‐by‐frame tracking of 34 cells through their cycle.

The analysis is based on manual recognition and recording of mitotic events within each time frame to ensure maximum accuracy. A typical image frame is shown in Figure 1 (N = 65 cells) and within it the mitotic cells are clearly differentiated as they detach from the surface of the culture well and round up prior to dividing. The evolving series of these events forms the starting point of the statistical analysis. A 10 h section of a sequence, measured from the A549 cell line, is shown in Figure 2A. Two aspects of the division process can be seen in this image: (i) the interevent times are to some degree random in length and (ii) there is an overall trend to an increasing rate of event occurrence with time. This is as expected, with the stochastic nature of the Poisson process producing varying time intervals and the deterministic process of cell proliferation leading to an ever increasing probability of the occurrence of a division event within a chosen time interval. The time dependent division rate translates to a time varying *λ* term within the event statistics and makes the process a nonhomogeneous Poisson (see Materials and Methods). In this case *λ*(*t*) is of an exponential form and processes of this nature have been well studied in relation to mortality, where the conditional probability of the occurrence of death doubles within a fixed time period 14, 15. Here, we see the same mathematical forms arising from an increasing probability of birth rather than death, driven by the growing cell population. A comparison of the measured interevent time distribution over a 40 h time period to the statistical prediction, using the nonhomogeneous Poisson formalism [Eqs. (5)], is shown in Figure 2B. The model accurately describes the data and indicates a mean intermitotic time, *t*_IMT_ = 21.1 ± 1.2 h (error bounds correspond to 95% confidence fit range). The experiment acquisition interval of 15 min sets the minimum resolution of interevent time and so for time steps in which multiple mitoses appear in the image, the interevent times are not specified but merely recorded as having a Δ*t* < 15 min. The accumulated number of division events is shown in Figure 2C and increases exponentially as expected. To validate the *t*_IMT_ value manual tracking of 34 cells from the point of birth through to division was undertaken to obtain a direct observation of the cell cycle time. The histogram of the measured values is shown in Figure 2D and shows a mean of 20.1 h with standard deviation of 4.2 h; thus the value determined from the intermitotic time distribution is well within the directly determined, value range. The results displayed in Figure 2 confirm one of the key aspects of the Poisson series analysis—information on the cell proliferation dynamics (i.e., *t*_IMT_) can be obtained through unreferenced observation of events, there is no requirement for identification or relation of cells. In general terms, the cell population is analysed via monitoring of process rather than components.

### Cell Synchronization

**Figure 3:**
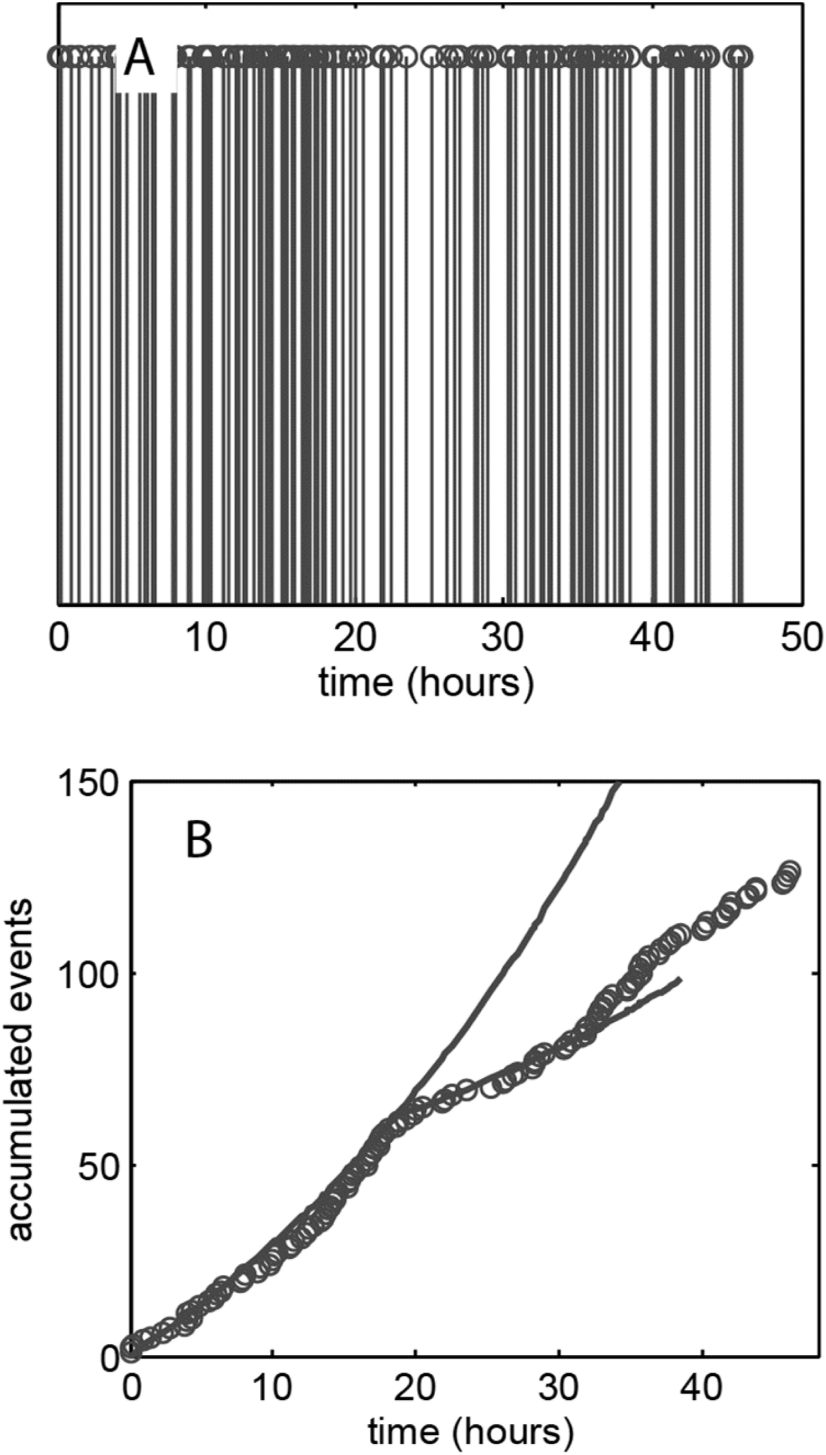
**A**: Mitotic event series for BEAS‐2B cells seeded at a density of 1.5 × 10^5^ cells ml^−1^. **B**: Accumulated count of cell division events (circles) with model predictions (solid line) based on exponential population growth. The model curves assume that 100% of cells undergo unsynchronized division between the 0 and 18 h time points and 30% do so between the 18 and 32 h time points.

**Figure 4:**
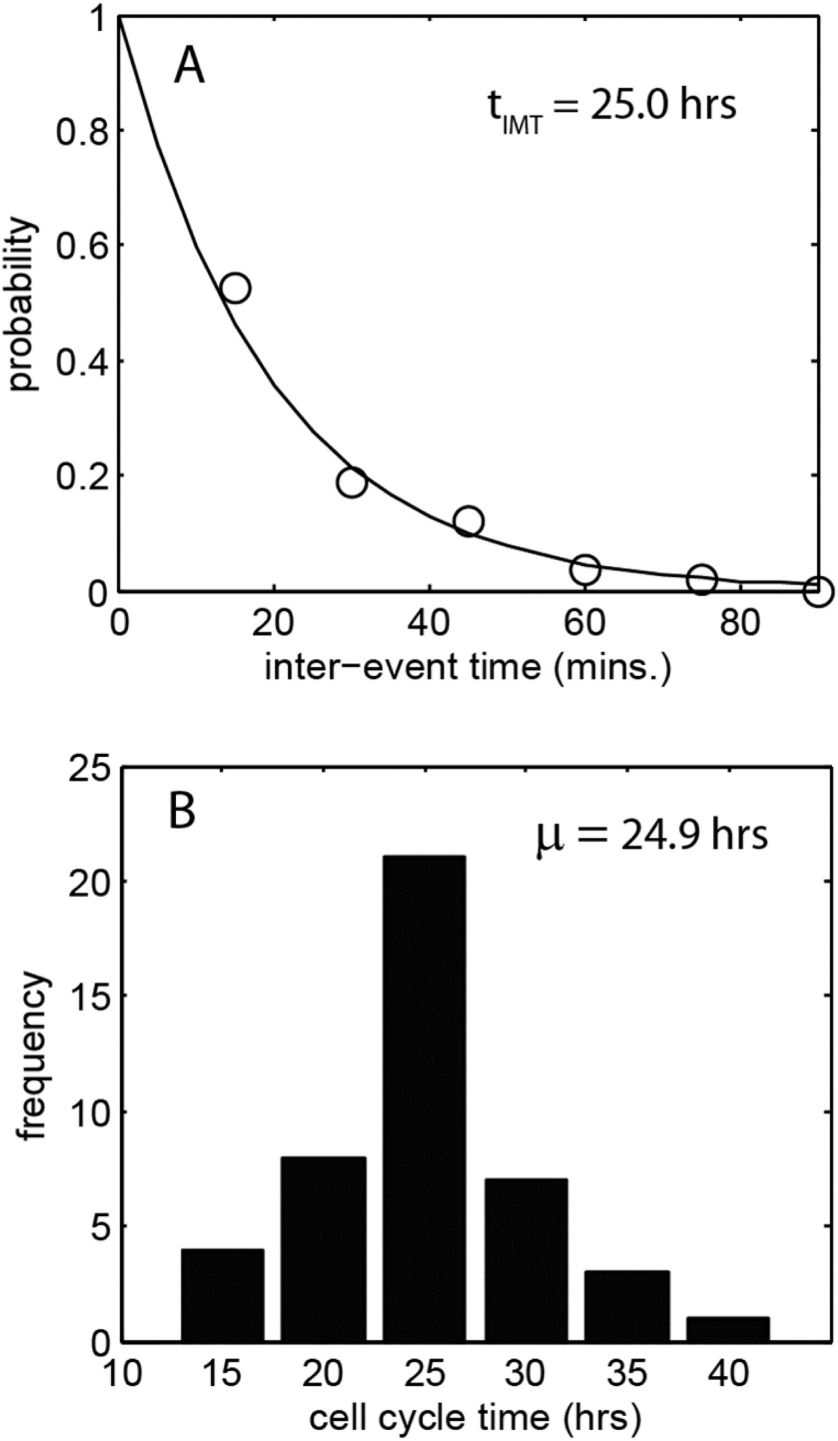
**A**: probability distribution of the interevent time for events shown in Figure 3A, occurring between the 0 and 18 h time points (circles – measurement data, solid line – statistical fit). The *t*_IMT_ value indicated is obtained by a best fit to the data assuming a nonhomogeneous poisson process. **B**: Histogram of measured cell cycle time obtained from manual, frame‐by‐frame tracking of 44 cells through their cycle.

The fundamental assumption of Poisson statistics is that the distribution of event occurrence is random and so synchronisation of cells within their life cycle will lead to a deviation of the measured data set from the model statistics. An example of this is shown in Figure 3, which shows the event time‐series and accumulated event curve, over a 48 h period, for the BEAS‐2B cell line. Synchronisation of cell cycle progression readily occurs within this cell line and this exhibits itself, between the 20 and 30 h time points, as an interval of sparse events in Figure 3A and a reduced rate of event accumulation in Figure 3B. Analysis of the intermitotic time for the initial exponential growth phase (0–18 h), under the assumption of complete cell cycle randomization, provides a *t*_IMT_ = 25.0 ± 1.1 h and this is within the range indicated by direct observation of cells through a full cycle (Figs. 4A and 4B). Thus the data is consistent with the situation in which an initially randomized population becomes partially synchronized during the cell culture, leading to a reduction in event occurrence between 20 and 30 hours. An estimate of the degree of synchronisation can be obtained from the data by fitting under the assumption of a fraction of the population obeying the nonhomogeneous Poisson behaviour (solid lines in Fig. 3B). This is implemented by taking a fraction of the calculated event occurrence over the 20 to 30 hour period [i.e., a fractional multiplier is applied to Eq. (2)]. The result indicates that only 30% of the population follows randomized division during the 20–30 h period, that is, 70% population synchronisation. We stress that the cycle synchronisation seen here is inherent to the cell culture process (see Supporting Information for further examples) and was not deliberately induced, indeed it was an unexpected aspect of the experiment. The ability to identify and quantify this from basic image acquisition, undertaken within the culture incubator, is a major benefit of the Poisson time series technique. Simple cell counts prior and postincubation would merely indicate a reduced average growth rate.

**Figure 5:**
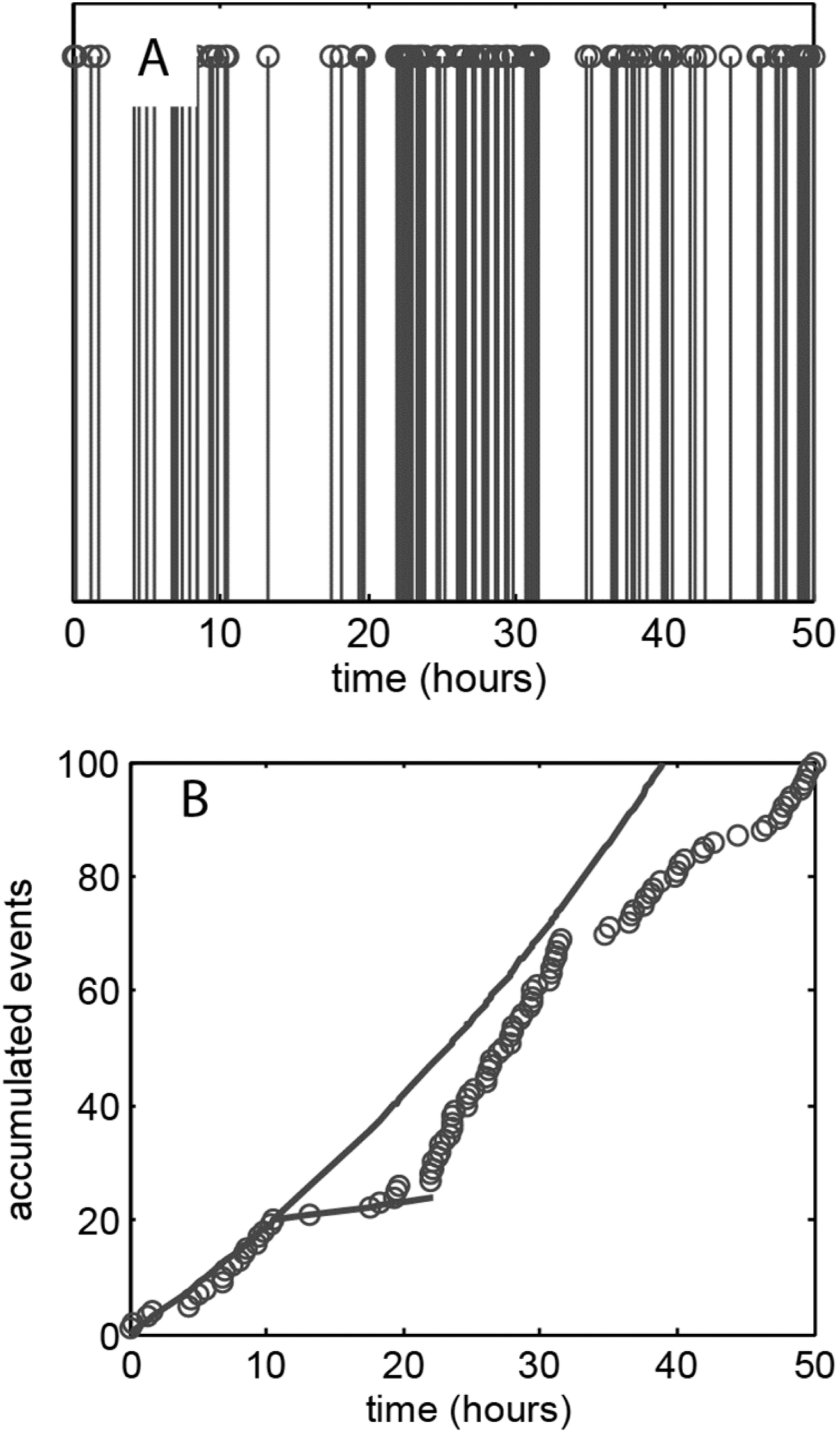
**A**: Mitotic event series for BEAS‐2B cells seeded at a density of 1.0 × 10^5^ cells ml^−1^ and serum starved for 72 h prior to data acquisition. **B**: Accumulated count of cell division events (circles) with model predictions (solid line) based on exponential population growth. The model curves assume that 100% of cells undergo unsynchronised division between the 0 and 10 h time points and 15% do so between the 10 and 21 h time points.

Controlled induction of cell cycle synchronisation through serum starvation (see Materials and Methods) enhanced the degree of synchronisation within the BEAS‐2B cell line, as shown in Figure 5. Analysis of cell proliferation in the 48 h following a 72 h serum deprivation period showed a marked absence of division events from 10 to 20 h following replenishment of the serum. Fitting of the data using Poisson time series analysis indicates that 85% of the population were synchronised in cell cycle. The signature of cell synchronisation can also be seen, one cell cycle time later, at the 32 h time point where there is a second region of reduced occurrence of mitotic events.

### Automated Image Analysis

**Figure 6:**
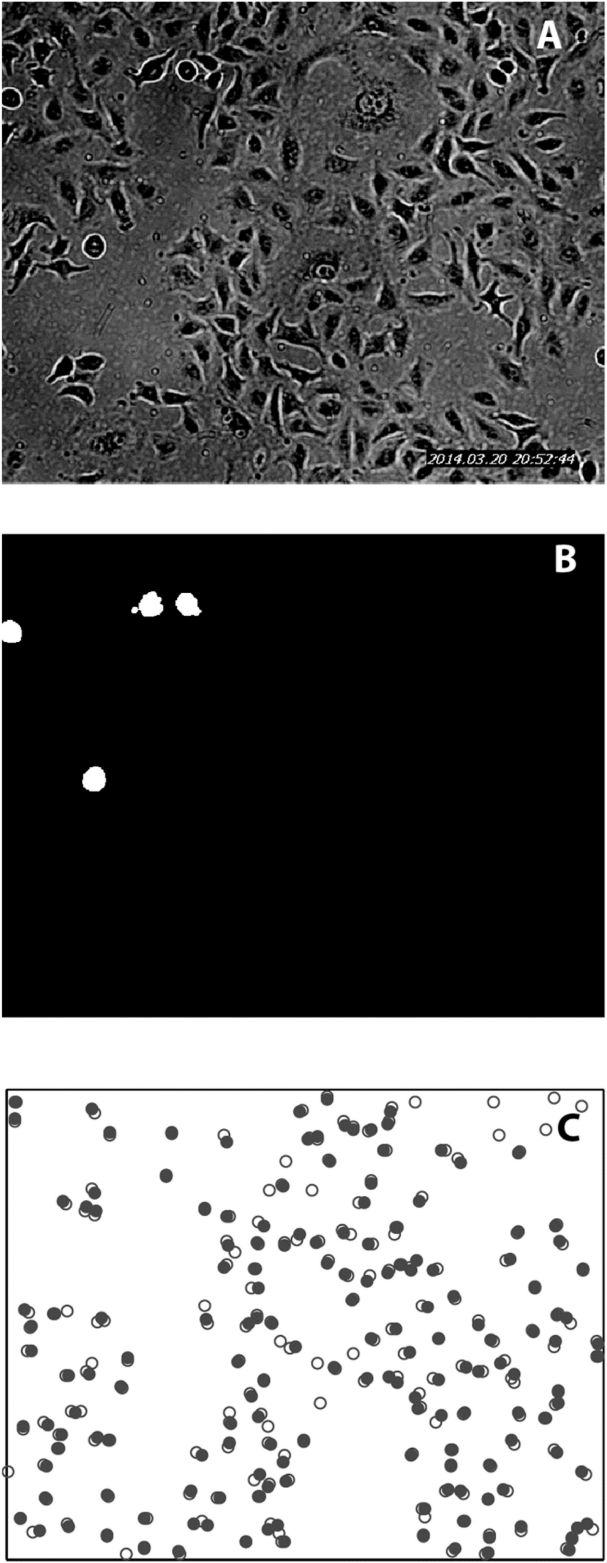
**A**: Representative bright field image of A549 cells, seeded at a density of 1.5 × 10^5^ cells ml^−1^. **B**: Mitotic cell identification mask generated from the image in Figure 6A. **C**: Plot of location of all division events shown in Figure 2C (open circles – manual identification, closed circles – automated identification using mitotic cell masks).

The simplified image processing required for the Poisson analysis allows ready automation of data acquisition using bright field images. In the frame, shown in Figure 6A, a number of mitotic events can be seen in the upper left quadrant of the image (image from time series for A549 cells shown in Figs. 2 and 3). These cells are easily identified due to their circular morphology, relatively dark shading and evidence of chromosome alignment across the centre of the cell. The results of automated detection and masking of these dividing cells are shown in Figure 6B; this processing was done using ImageJ software and involved simple image inversion, filtering and mask selection based on size and circularity (see Supporting Information for full details). Running the ImageJ script for all 160 frames across the 40 h measurement period provides fully automated acquisition of the mitotic events. These are shown in Figure 6C together with the manually identified data set. The automated process successfully identifies 87% of the mitotic events, a level of accuracy which incurs only a minor difference in the calculated *t*_IMT_, which is calculated to be 21.6 h from the interevent time series (manual identification of events gives *t*_IMT_ = 21.1 h).

### Application Within a Scratch‐Wound Assay

**Figure 7:**
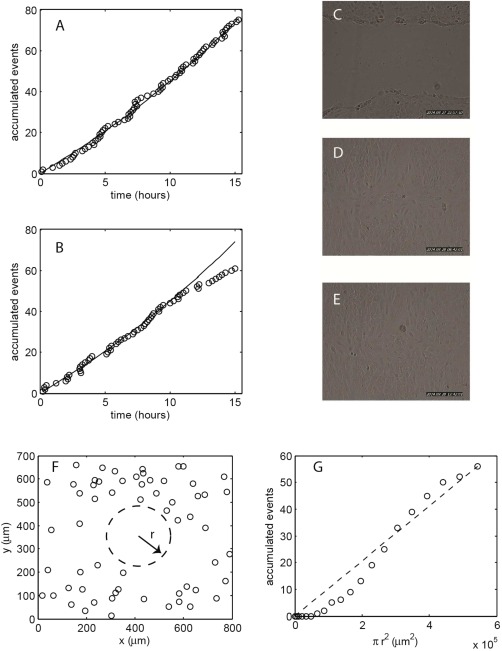
**A**: Accumulated mitotic events for BEAS‐2B cells over a 15 h period prior to a scratch assay. **B**: Accumulated mitotic events for the 15 h immediately following creation of a scratch in the cell layer. **C**–**E**: Image frames acquired at 0, 10, and 15 h following creation of the scratch. **F**: Spatial map of the mitotic events shown in B. The dashed circle indicates the position of the variable area used for counting of spatial events. **G**: Spatial accumulation of mitotic events shown in B.

The scratch‐wound assay is a common tool for studying cell migration characteristics 16. In this work we choose the assay to demonstrate application of the Poisson analysis technique, within a commonly used protocol, for which temporal and spatial cell information is important. Note that our aim here is not to undertake a detailed biological study but rather to simply show the technique within the context of a specific assay. BEAS‐2B cells were incubated over a 48 h period to form a confluent layer on the culture dish surface, a pipette tip was then used to scratch across the surface and so remove cells from a given area. The cells were then cultured for an additional 48 h, during which they recolonized the scratched area. Continuous monitoring of the cell population during the assay by the JuLI microscope allowed us to analyse the temporal and spatial correlation of mitoses and to relate these measurements to the cell migration that fills the scratch space. The accumulation of mitotic events over time, prescratch and postscratch, is shown in Figures 7A and 7B. The data confirms exponential population growth during the establishment of the confluent layer with division rates similar to that seen in other experiments with this cell line. In the 10 h immediately following creation of the scratch there is again an exponential growth in the cell number, of a similar magnitude to that seen prior to the scratch. However, at longer times (10–15 h) there is a slowdown in the accumulation of mitoses indicating a reduced rate of population growth. Inspection of the image set shows that cell migration recolonizes the scratch area within the 10–12 h time range (Figures 7C–7E). Thus, it would seem that the slowdown in population growth is linked to the spatial organisation of the cell colony. Quantitative assessment of the spatial coordination of cells is also provided by implementation of the Poisson analysis to a mitotic event series, but this time the occurrence of mitoses is tracked as a function of increasing area rather than increasing time. A map of all the mitoses occurring within the 15 h following the scratch (data in Fig. 7B) is shown in Figure 7F. By applying the Poisson analysis to this data, we plot the number of accumulated mitotic events as a function of increasing area (Fig. 7G). The count is taken for events within a circle of increasing radius, placed at the centre of the image (*x* = 400 μm, *y* = 350 μm). The dashed line in Figure 7G shows the expected behavior for a Poisson process: a linear increase in event number with increasing area according to a mean rate of occurrence, *λ*. The data shows a substantial deviation from this line for small area, indicating sparse events, in this case due to the removal of cells from the center of the analysis area by the scratch. Here, the general trends shown by the Poisson analysis are unsurprising as they are predetermined by the specific conditions of the assay. Nonetheless, the demonstration does show how quantitative information can be obtained on the spatial relationship of mitotic events, thus allowing discrimination of random cellular division from synchronised behaviour across a colony.

## Discussion

Poisson statistics describe the probability of events within a stochastic series, providing estimates of their spacing in time under the assumption of independence of any given event from previous instances. The focus on the “event,” that is, the outcome or visible expression of a process, makes this probability theory well suited to applications in cell proliferation studies. The evolution of a cell population is manifest in the cell division events which drive it and so these provide a measurable descriptor of the population dynamics. The question of whether cell division is asynchronous in space and time across a cell colony is also pertinent and so statistical assessment of the randomness of a sequence of cell division events is a powerful analytical tool. In addition to providing a mathematical framework for investigation, the Poisson analysis also simplifies the experiment protocols. The study of cell population dynamics proceeds from recognition of cell level events without need for recognition of the cells as the cell cycle processes are quantified by the discrete points at which division occurs rather than continuous monitoring of the whole system. The mathematical framework of Poisson statistics is also flexible and wide‐ranging enough to encompass diverse or altered biology. We show here how an extension to a nonhomogeneous Poisson series allows consideration of time varying processes with the specific example of the exponential form which is typical of a Gompertz process 14. Other functional dependencies can be readily accommodated by using the Weibull family of probability distributions which are based on power law descriptions of the Poisson rate parameter 17. Spatial inhomogeneity of biological processes, for example, arising from phenotype variation, can also be accommodated through the use of compound Poisson distributions such as the Overdispersed Poisson or the equivalent Negative Binomial distribution 18. In fact these statistical descriptions have been widely used in biology to describe local nonuniformity of a range of ecological species 19, 20, 21.

The demonstration of the technique described here provided accurate quantification of cell cycle time of in‐vitro cultures and for BEAS‐2B cells highlighted hitherto unsuspected temporal synchronization of the population. Implementation is straightforward, allowing automated image analysis of bright field frames acquired with a microscope of modest performance. The ability to acquire detailed data on cell proliferation dynamics, without the need for high performance microscopy, also provides benefits beyond simplified data acquisition. The use of “low‐level” optics allows “high‐level” biological control as the imaging instrument becomes simple enough to operate inside a cell incubator. This provides a measurement approach in which the biology of the cells can become the primary concern of the researcher without the hindrance of complex imaging requirements, that is, accurate control of the cellular environment can be achieved within a dedicated system rather than integrated into the microscope.

## Supporting information

Supporting Information
