# Supplementary material for: Poisson‐event‐based analysis of cell proliferation
Source: Cytometry A. 2015 Jan 8;87(5):385–92. doi: 10.1002/cyto.a.22620 (PMC4964947; doi:10.1002/cyto.a.22620)
Supplement: Supplementary file 1 — Supporting Information [file CYTO-87-385-s001.docx]

**Supplementary Information: Poisson-event based analysis of Cell Proliferation**

**Huw D. Summers, John W. Wills, M. Rowan Brown and Paul Rees**

Supplementary figures –

1. Analysis at different cell seeding densities

2. Analysis of serum starvation experiments

Supplementary information –

Details of ImageJ processing protocol used for automated detection of mitotic events in bright field images.

**Supplementary figure, S1**

BEAS-2B 1x10^5^ cells ml^-1^,

t_IMT_ = 31.1 hrs

BEAS-2B 1.5x10^5^ cells ml^-1^,

t_IMT_ = 28.4 hrs

BEAS-2B 1.5x10^5^ cells ml^-1^ (Repeat),

t_IMT_ = 40.7 hrs

BEAS-2B 2x10^5^ cells ml^-1^,

t_IMT_ = 29.3 hrs

BEAS-2B 2x10^5^ cells ml^-1^ (Repeat),

t_IMT_ = 44.1 hrs

A549 1x10^5^ cells ml^-1^ ,

t_IMT_ = 25.3 hrs

Each plot represents a separate cell culture and image acquisition experiment run. The t_IMT_ values are calculated from the initial exponential growth phase at the start of each data set (dashed red line). The marked variability in the t_IMT_ values appears to be related to the degree of cell synchronicity with highly synchronised populations showing a reduced t_IMT_ in their initial growth phase. The solid red line is a reference curve indicating linear growth.

Event count

Event count

Time (hrs)

Time (hrs)

Time (hrs)

Time (hrs)

Time (hrs)

Time (hrs)

**Supplementary figure, S2**

BEAS-2B serum starve I – depletion phase

1x10^5^ cells ml^-1^, t_IMT_ = 28.5 hrs

BEAS-2B serum starve II – depletion phase

1x10^5^ cells ml^-1^, t_IMT_ = 32.9 hrs

BEAS-2B serum starve II – recovery phase

BEAS-2B serum starve I – recovery phase

Depletion phase – cells cultured without serum

Recovery phase – cells cultured with full serum complement

**Supplementary Information - ImageJ protocol for automated detection of cell division:**

The following protocol was applied to all images within a given time series.

1. *Edit* - INVERT image

Creates a negative image in which mitotic cells appear as bright spots.

1. *Process – filters* – MAXIMUM (3 pixel setting)

Merges neighbouring pixels by setting them to the maximum value, this broadens out the bright spots of the dividing cells.

1. *Image – adjust* – BRIGHTNESS/CONTRAST

Optimised to enhance the contrast of the mitotic cells relative to the image background.

1. *Process – binary* – MAKE BINARY

Create a binary mask.

1. *Analyze* – ANALYZE PARTICLES (400-infinity pixels^2 size, 0.4 – 1 circularity)

Filter out mitotic cells from non-mitotic by size and circularity.

1. Post processing in MATLAB – simple screening algorithm applied to count particles within each frame and to ignore multiple records due to the division process potentially occupying 2-3 sequential, 15 minute interval, frames.
